# Untargeted Metabolomics Analysis Based on LC-QTOF-MS to Investigate the Phenolic Composition of Red and White Wines Elaborated from Sonicated Grapes

**DOI:** 10.3390/foods13111761

**Published:** 2024-06-04

**Authors:** Alejandro Martínez-Moreno, Paula Pérez-Porras, Ana Belén Bautista-Ortín, Encarna Gómez-Plaza, Fernando Vallejo

**Affiliations:** 1 Department of Food Science and Technology, Faculty of Veterinary Sciences, University of Murcia, Campus de Espinardo, 30100 Murcia, Spain; martinezamoreno@gmail.com (A.M.-M.); paula.perez2@um.es (P.P.-P.); anabel@um.es (A.B.B.-O.); 2 Metabolomic Platform, Centro de Edafología y Biología Aplicada del Segura (CEBAS-CSIC), Campus Universitario de Espinardo, 30100 Murcia, Spain

**Keywords:** sonication, wines, bioactive compounds, untargeted metabolomics, LC-MS

## Abstract

Ultrasounds are considered an emerging technology in the wine industry. Concretely, in 2019, the International Organization of Vine and Wine (OIV) officially approved their use for the treatment of crushed grapes to increase the level of phenolic compound extraction. The main objective of this study was to validate an untargeted metabolomics approach as an analytical tool for identifying novel markers associated with sonication. To do so, the influence of a sonication treatment on the metabolic profile was studied in four typically commercial varietal wines, i.e., two red wines from ‘Syrah’ and ‘Cabernet Sauvignon’ grapes and two white wines from ‘Macabeo’ and ‘Airén’ grapes. A robust classification and prediction model was created employing supervised techniques such as partial least-squares discriminant analysis (PLS-DA). The findings indicated that the grapes subjected to high-power ultrasound conditions experienced cell wall disruption due to the cavitation phenomenon, resulting in significant changes in various phenolic compounds (including hydroxycinnamic acids and flavonoids) present in these wines compared to wines from non-sonicated grapes. Additionally, new metabolites were tentatively identified through untargeted metabolomics techniques. This study represents the successful application of the untargeted metabolomics approach employing a UHPLC-QTOF system to discern how grape sonication affects bioactive secondary metabolites in wines.

## 1. Introduction

Wine is one of the most complex food matrices in existence. Along with a wide variety of other compounds, phenolic, aromatic and polysaccharide compounds mainly play elementary roles in the organoleptic characteristics of wines, such as color, aroma or texture, and that is why, nowadays, oenological research and the related industry have intensified their efforts in the use of new technologies that allow for obtaining wines of higher quality than before and with more appreciated color and aroma profile.

As a result, the use of technologies such as high hydrostatic pressures, pulsed electric fields or ultrasound in the oenological industry has been of great interest in last years [1], and their industrial application was recently approved by the International Organization of Vine and Wine (OIV) [2].

Of particular interest among these technologies is the use of high-power ultrasound. This technology is a non-thermal processing technology, relatively economical, non-hazardous, and respectful of the environment—as it does not use organic solvents [3,4]—commonly applied in food industries for various purposes [5,6].

These mechanical waves with a frequency higher than 20 kHz have been widely studied in their application to obtain bioactive compounds in wine from grapes, focusing especially on the intensification of the extraction of phenolic, aromatic and polysaccharide compounds that improve the antioxidant capacity and the organoleptic quality of the wines produced [1,7]. This effect is obtained due to the depolymerization, through the cavitation process generated by ultrasound, of cell wall structures that act as a limiting barrier to the extraction of the compounds of interest [8]. During periods of wave compression and rarefaction, bubbles that constantly increase and reduce in size are generated. In transient cavitation, these bubbles acquire an increasingly larger size until they reach a critical size at which point, they collapse and implode. In this process, large amounts of energy are released, reaching temperatures of 5000 K and pressures of 200 MPa, as well as shock waves in the form of microjets of liquid, capable of breaking nearby solid structures [8].

In this sense, the efficacy of high-power ultrasound in rupturing the cell wall of skin cells has been demonstrated [9,10], with its consequent release of compounds of interest that are analyzed in wine (phenolic compounds, polysaccharides and aromatic compounds) considering the structural and compositional differences of cell walls depending on the grape ripening stage [11,12], microbiological alterations [12] and varietal nature [10]. Thus, conclusive results have been obtained from the optimization of the process of making value-added red wines through the use of US and usual processing times [10,11,13,14], as well as quality red, white and rosé wines with the use of US and shorter processing times [9,15,16,17], especially in those cases where US is combined with traditional techniques such as the addition of pectolytic enzymes [11,18], thus allowing not only for the improvement of the organoleptic characteristics of those wines whose initial potential is insufficient, but also for the reduction of the processing time without detriment to quality, increasing the productive capacity of wineries.

Nevertheless, it is well known that wine is a complex matrix with hundreds of compounds from different chemical families, and the majority of studies that can be found in the literature focus on the analysis of specific compounds [19] and do not allow for a holistic and integrative perspective that reliably represents the effect of ultrasound or other technologies on the treated products. In this sense, the growing interest in the “omics approach”, which has been little studied in the wine field, is being decisive [20]. Metabolomics is the scientific study of small molecules, commonly known as metabolites, based on a complete chemical analysis in order to detect and quantify as many substances as possible [21]. In oenology, its use has raised great interest in recent years to guarantee not only the quality of wine but also its traceability, by detecting authenticity and adulteration, determining varietal and geolocation (*terroir*) fingerprints and even controlling production processes such as fermentation [22].

To carry out metabolomic analysis, a great diversity of techniques are used, both for the separation of compounds (liquid or gas chromatography being the most used technique in oenology) and for their determination, such as nuclear magnetic resonance or mass spectrometry, being this last one especially used for this objective. Currently, numerous types of mass spectrometers with a very high degree of sophistication depending on their resolution and mass precision are available to carry out the separation and detection of the generated ions, especially useful in wine-omics, QTOF and Orbitrap detectors.

Metabolomic analyses have been generally classified as targeted or untargeted. Targeted analyses focus on a specific group of metabolites, in most cases aiming at the identification and quantification of as many metabolites as possible within the group [23], and require a high level of purification and the selective extraction of metabolites. However, as purified compounds are not always available, libraries are used to check similarities, although unfortunately, these mass spectral databases lack a large number of metabolites, which prevents the comprehensive understanding of metabolic systems. In fact, until a few years ago, 62% of the masses found by untargeted analysis with the implementation of high-resolution spectrometers in wine-omics could not be identified using the databases [22], which has resulted in a profound lack of knowledge of the wine matrix. In contrast, untargeted (also known as comprehensive) metabolomics focuses on the detection of as many groups of metabolites as possible to obtain patterns or fingerprints without necessarily identifying or quantifying specific compounds [24], which is of special interest in complex matrices such as wine. As an example, in addition to Alañón et al. [22], Pinu [25] reviewed the different works conducted with the use of both targeted and untargeted metabolomics to study grapes and wines and showing the tremendous potential of metabolomics. Also, Lloyd et al. [26] reviewed and explored different metabolomics approaches that were successfully employed to comprehensively understand the chemical diversity of grapes, yeast and wine.

In this sense, given the chemical diversity of metabolites present in wine and considering the interest in US technology for the extraction of compounds of interest from grape and must during winemaking, the analysis of secondary metabolites, such as phenolic compounds, in wine based on untargeted metabolomics may be a potential tool to discriminate between different types of oenological processes.

The novelty of this work lies in the proposal of a fast and effective approach (i.e., phenolic profiling with supervised multivariate PLS-DA) to screen and evaluate the wide phenolic composition and possible metabolomic markers in red and white wines of different varieties based on the effects generated by ultrasound during winemaking.

## 2. Materials and Methods

### 2.1. Wine Sample

Samples of red wine from two different grape varieties were used for the analysis, i.e., ‘Syrah’ (S) and ‘Cabernet Sauvignon’ (CS), as well as samples of white wine from two grape varieties, i.e., ‘Airén’ (A) and ‘Macabeo’ (Mac), which were obtained at the time of bottling of the wines, once their production was completed.

### 2.2. Winemaking and US Treatment

For each variety, two types of wine were developed using two procedures: a control procedure and another involving an US treatment. The control procedure for the red wines consisted of crushing and destemming the grapes and a fermentative maceration process in 50 L stainless-steel tanks for 7 days at a controlled temperature (24 ± 2 °C), with total acidity corrected to 5 g/L and the addition of selected *Saccharomyces cerevisiae* yeast strains (Viniferm CT007, Agrovin, Alcázar de San Juan, Spain) at 30 g/hL. After 7 days of maceration, the solid parts of the grapes were devatted and pressed using a 75 L pneumatic press, and alcoholic fermentation continued in the tanks. Once the fermentation was finished, the first racking, sulfiting and cold stabilization took place in a chamber at 2 °C for a month. After that, a second racking and bottling took place.

For the preparation involving the US treatment, prior to fermentative maceration, the crushed and destemmed pulp was subjected to US treatment using the Ultrawine device (Agrovin SA, Alcázar de San Juan, Ciudad Real, Spain). This device is equipped with two hexagonal sonoreactors with several sonoplates attached. It worked at a frequency of 30 kHz, a power of 9000 W and power density of 58.5 Wcm^−2^ and 540 WL^−1^. The design of the US equipment and the low residence time of the must in the system allowed the must temperature to be maintained.

In the case of the white wines, the control process consisted of crushing and destemming the grapes, directly pressing the solid parts of the grapes and racking the must with the addition of the Enozym Lux enzyme (Agrovin SA, Alcázar de San Juan, Ciudad Real, Spain), which was left to act for 24 h, after which, the total acidity was corrected to 5.5 g/L. Selected *Saccharomyces cerevisiae* yeast strains were added for the development of alcoholic fermentation (Viniferm BY, Agrovin SA, Alcázar de San Juan, Ciudad Real, Spain) that took place in a temperature-controlled chamber (18 ± 2 °C). When it ended, the wines were racked, sulfited and cold-stabilized in a temperature-controlled chamber (2 °C), after which, the wines were filtered and bottled. For the US procedure, the treatment was carried out after crushing and before pressing, using the same conditions as for the red wines.

### 2.3. Wine Untargeted Metabolomics Analysis by UPLC-QTOF

Triplicate wine samples were centrifuged at 10,480× *g* for 10 min, and each supernatant was filtered through a 0.45 µm cellulose nitrate membrane filter (Agilent Technologies, Waldbronn, Germany). Non-targeted metabolomics was approached as previously reported using an Agilent 1290 Infinity series LC system coupled to a 6550 I-Funnel Accurate-Mass QTOF (Agilent Technologies, Waldbronn, Germany) with a dual electrospray ionization interface (ESI-Jet Stream Technology, Waldbronn, Germany). The samples were injected into a reverse-phase Poroshell 120 EC-C18 column (Agilent) following the operating conditions described by Gomez-Bellot et al. [27]. As there were no specific target analytes (untargeted analysis), generic settings were applied to obtain as many compounds as possible. QTOF-MS offers high selectivity, sensitivity, resolution and mass accuracy, providing a powerful tool for complex sample metabolic profiling. All samples were injected in the same batch, in a randomized order to avoid sample bias. A mixture with one replicate of each group of samples was used as ‘quality control’ and was injected at the beginning and at the end of the batch [27].

Detection was performed in both positive and negative ionization modes, and the data were acquired using the Mass Hunter Workstation software (version B.08.00, Service Pack 1, Agilent). Feature extraction statistical analysis and tentative identification were carried out according to previous publications [27,28].

### 2.4. Data Treatment. Multivariate Model Analysis

The data matrix was exported to Mass Profiler professional (MPP, Agilent technologies, Waldbronn, Germany) for data management. The data matrices were processed with log transformation and auto scaling prior to univariate and multivariate analysis [28]. Partial least-square discriminant analysis was used to check the classification of the sample groups looking for group differences, while the VIP (variable importance in projection) score provided information on the importance of the entities. All univariate analyses were performed with the MPP software, after the multivariate analysis evaluation. The final list of features was used for metabolite identification with the purchased METLIN databases [28].

## 3. Results and Discussion

**Figure 1:**
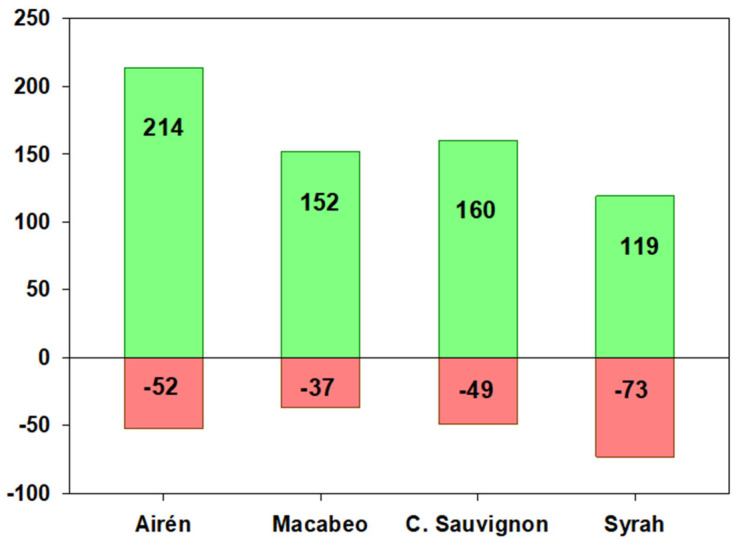
Increase or decrease in the number of metabolites in wines made from sonicated grapes compared to their respective control wines made from non-sonicated grapes.

The total metabolites with significant differences in levels obtained from the wine samples through untargeted metabolomic analysis by UPLC-QTOF are shown in Figure 1. This Figure shows the increase or decrease in the number of metabolites in wines made from ultrasound-treated grapes compared to their respective control wines. An increase of 214 and 152 metabolites in wines from ultrasound-treated grapes of the ‘Airén’ and ‘Macabeo’ varieties was observed, along with a decrease of 52 and 37 metabolites.

For the red wines, an increase of 119 and 160 metabolites in ‘Syrah’ and ‘Cabernet Sauvignon’ was obtained. Overall, the results pointed to an effect of high-power ultrasound (US) on grape tissue disruption and consequently on the concentration of the extracted metabolites during maceration. This effect was observed by Pérez-Porras et al. [9,10], who observed cellular alteration, through the plasmolysis of subepidermal cells and the collapse of the mesocarp of skin cells, in addition to a greater number of cells without coloration, indicating the release of intracellular content into the medium [9]. We observed an improvement in color intensity and the total polyphenol index for the wines obtained from the sonication of grapes of the different red varieties ‘Monastrell’, ‘Syrah’ and ‘Cabernet Sauvignon’ [9,10].

Although all varieties showed an increase in total metabolites, the white varieties ‘Airén’ and ‘Macabeo’ showed a higher ratio of increased than reduced metabolites, with a 4.1-fold increase for each variety. Among the red varieties, ‘Cabernet Sauvignon’ showed a 3.2-fold increase in the number of metabolites. As stated before, numerous studies have demonstrated the effectiveness of high-power ultrasound application at an industrial level to enhance the quality of red wines, attributed to the increase in secondary metabolites of interest such as phenolic compounds [10,29]. However, to the best of our knowledge, there are no studies on the effect of high-power ultrasound utilization in white varieties and its impact on the metabolic profile of the resulting wines at an industrial scale. On the other hand, the majority of the studies carried out to date focused on the analysis of specific compounds such as tannins, anthocyanins or polysaccharides [14,30], especially in red wines, and of aromatic compounds and amino acids in white wines [13,15], making it necessary to undertake more in-depth studies on the effect of US on the extraction of other types of metabolites.

**Figure 2:**
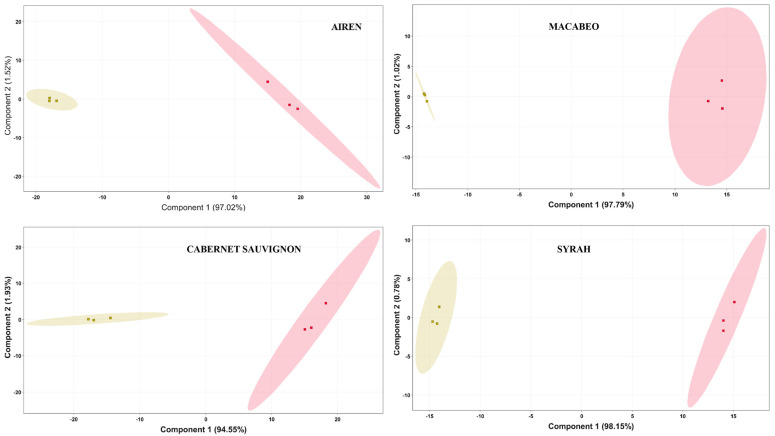
PLS-DA model of the full data set. Yellow dots: control wines (from non-sonicated grapes); red dots: wines from sonicated grapes.

To illustrate how the metabolites detected can really indicate that the US treatment clearly led to significant different wines, a PLS-DA model of the final data matrix was created by MPP software to evaluate the classification of the samples from the white and red varieties into groups (Figure 2). The pre-processing operations provided a data matrix based on 925, 948, 859 and 808 metabolites from the data sets of ‘Airén’, ‘Cabernet Sauvignon’, ‘Macabeo’ and ‘Syrah’, respectively. The calculated PLS-DA model, based on six samples and three components for each group, showed a variance from 99.0% to 99.6% (R2 ranged from 0.990 to 0.996) according to the cross-validation prediction (Q2 ranged from 0.801 to 0.859). The first principal component PC1 explained more than 90% of the variance for all wines of the four studied varieties. It is important to note that the high percentage of variability explained by the first principal component indicated that there was a clear separation between the wines in the space defined by this component. This indicated that the control wines and those made from ultrasound-treated grapes were very different from each other in terms of the metabolites measured in the analysis. These results confirmed the previously observed great difference between wines from sonicated grapes and their controls, for both red and white varieties. Pérez-Porras et al. [12], in an industrial grape sonication study, also observed significant differences between sonicated and control ‘Syrah’ red wines. In fact, of the three factors that were studied in this work (sonicated, ripening and sanitary status of the grapes), sonication was the factor that led to the greatest separation, with a PC of 47% of the total variability. Pérez-Porras et al. [10] also observed a large difference between sonicated and control red wines from ‘Monastrell’ grapes. Therefore, this study showed the effectiveness of sonication in changing the metabolomic profile of wines, suggesting that sonication may be considered an innovative and promising technology for the wine industry to improve the quality of wines.

However, although non-targeted metabolomics analysis is a highly valuable tool for obtaining a broad view of treatment effect patterns or determining biomarkers, it has an important limitation linked to the scarce information that is currently available for the identification of the differential metabolites in different samples. The multivariate analysis indicated that the ultrasound treatment led to significant differences in 1067 metabolites, (*t*-test unpaired; corrected *p*-value cut-off: 0.05; *p*-value computation: asymptotic; multiple testing correction: Benjamini–Hochberg). Combining the higher VIP values obtained, the most statistically significant entities and the most accurate matches in the databases, a list of 14 metabolites whose levels increased in wines made from sonicated grapes compared to the control wines were tentatively identified [31] (Table 1 and Table 2).

Three hydroxycinnamic acids (in combined or free form) were identified in the ‘Airén’ variety (Table 1): caftaric, coumaric and p-coutaric acids. Hydroxycinnamic acids (especially their tartaric esters) are naturally present in grapes (skin and pulp) and are the most important phenolic compounds in grape pulp and white wines [32]. Their contents vary widely with the grape growing conditions, but usually, in white wines, hydroxycinnamic acids account for approximately 50% of the total phenolic content [33]. In addition to the high antioxidant capacity and anti-inflammatory properties attributed to hydroxycinnamic acids [34], which makes their consumption is of interest, they are also determinants of the organoleptic properties of the products in which they are found. Thus, they have been related to the texture of wines (including bitterness, astringency and viscosity) as well as to greater varietal character and complexity and to lower acidity [33,35,36].

However, hydroxycinnamic acids are also precursors of volatile phenolic compounds [37] that can significantly affect the sensory profile of wines. Moreover, it is known that these compounds are very susceptible to oxidation. When hydroxycinnamic acids are exposed to the free radicals present in the environment, they can undergo an oxidation reaction that leads to the formation of quinones. These quinones are responsible for the darkening or browning of wine and can contribute to its qualitative decay [38]. The oxidation process can alter the wine’s color, aroma, and taste, leading to a loss of freshness and vitality. Additionally, these reactions can lead to the appearance of undesirable flavor notes, negatively impacting the sensory quality of wine. Therefore, in the production of white wines from sonicated grapes, it would be necessary to implement appropriate winemaking practices during vinification and wine storage, such as the use of antioxidants, the control of oxygen exposure, and the maintenance of proper storage conditions, such as cool temperatures and absence of ultraviolet light.

Regarding the other white variety, ‘Macabeo’, five metabolites were tentatively identified, i.e., two hydroxycinnamic acids—coumaric acid and p-coutaric acid (both also identified in ‘Airén’)—two stilbenes—resveratrol and resveratrol 4′-glucoside—and one non-flavonoid phenol—hydroxytyrosol 1-O-glucoside. Stilbenes are bioactive compounds with a great range of biological activities potentially beneficial for human health, such as antioxidant, anti-tumor, anti-obesity and neuroprotective effects [39]. Grapes and wines are among the major sources of stilbenes. These compounds are located essentially in the skins of grapes, although it has also been reported that they may be present in grape seeds and stems [40].

As shown by the results, the levels of resveratrol (the major stilbene in grapes) and resveratrol 4′-glucoside increased significantly in wines made from sonicated grapes compared to their control wines. Some authors studied the diffusion of stilbenes from grapes to must/wine during the maceration process, observing that the transfer rates of stilbenes to wine are very low [41], leading to their maximum concentration after 12 days of maceration [42]. Therefore, the sonication of grapes can be a useful and effective tool to accelerate the diffusion and transfer of phenolic compounds such as stilbenes to wine, increasing their concentration and reducing the maceration times. Another identified metabolite that increased significantly in wines produced with ultrasounds is hydroxytyrosol-1-O-glucoside. Several health-enhancing activities (anticarcinogenic, cardiopreventive and antimicrobial properties) have been attributed to hydroxytyrosol [43]. This compound is one of the most potent antioxidants that we can find in nature and is mainly found in olive oil [44], although it has also been described in wines [45]. In 2011, due to its antioxidant properties, it was included by the European Food Safety Authority (EFSA) in the European food consumption database as a protective compound against oxidative damage [46]. Hydroxytyrosol is formed from the hydroxylation of the aromatic ring of tyrosol, which is a secondary metabolite derived from tyrosine formed by yeasts during alcoholic fermentation [47]. At the same time, the synthesis of tyrosol was described as directly proportional to the quantity of amino acids present in must [48]. Therefore, the significant increase in hydroxytyrosol 1-O-glucoside in wines made from sonicated grapes could be related to the increase in the concentration of amino acids in the sonicated must, as Carrera et al. [49] observed for white varieties, caused by the greater degradation and rupture of the cell walls of the grapes due to sonication [10]. Regarding the red grape varieties (Table 2), in ‘Syrah’, the sonication of crushed grapes led to an increase in only one metabolite, i.e., epicatechin 8-C-galactoside; a significant increase in this metabolite was observed in the wines produced with the ultrasound treatment.

This tentative metabolite is a monomeric form of flavanols (flavan-3-ols) found in grapes (skin and seed). Flavanols have a very important role in the stabilization of color in red wine during the aging process [50] and play a key role in determining the sensory properties of red wines, mainly astringency and bitterness [51]. The increase in epicatechin 8-C-galactoside levels in wines made from sonicated grapes may indicate a greater extraction of flavanols during maceration due to sonication [52]. In a study in which high-power ultrasound was applied to crushed ‘Monastrell’ grapes, Martínez-Pérez et al. [30] reported higher astringency of wines produced with ultrasounds compared to control wines. However, in another study involving ‘Syrah’ grapes at various ripening stages subjected to sonication, the authors did not observe a significant increase in the astringency or bitterness levels in wines made from the treated grapes compared to their controls [12]. Finally, an increase in ten metabolites was observed for the red variety of ‘Cabernet Sauvignon’ wine, including epicatechin 8-C-galactoside, resveratrol 4′-glucoside, quercetin-3′-glucuronide, epicatechin 3-O-gallate, epigallocatechin 3-(4-methyl-gallate), isorhamnetin 3-sophoroside, quercetin 3-galactoside, quercetin 3-(2″,3″,4″-triacetylgalactoside), resveratrol and kaempferol 3-(2″,3″-diacetyl-4″-p-coumaroylrhamnoside). Of the metabolites named above, five are phenolic compounds belonging to the flavonol family. Flavonols are a class of flavonoid compounds found in the berry skins of *Vitis vinifera* L. white and red grapes [53]. In red wine vinification, flavonols are extracted from the grape skin and pulp and transferred to the must/wine during the maceration period, determining the quality of the resulting wines. For this reason, one of the aims of the wine industry is to accelerate this extraction process during fermentation to reduce the maceration times.

High-power ultrasound, whose industrial application was authorized by the OIV in 2019 [54], has shown to be an effective technique for increasing the extraction of phenolic compounds and reducing the maceration times [55]. The results obtained in this work confirmed how the use of ultrasound technology significantly increased the levels of flavonols (quercetin-3′-glucuronide, isorhamnetin 3-sophoroside, quercetin 3-galactoside, quercetin 3-(2″,3″,4″-triacetylgalactoside) and kaempferol 3-(2″,3″-diacetyl-4″-p-coumaroylrhamnoside) in wines made from sonicated grapes compared to those from non-sonicated ones. The presence of flavonols in wine is of great interest due to their role as copigments in the color stabilization processes in young and aged wines [56]. One of the main effects of copigments is the increase in wine absorbance at 520 nm [57], the wavelength at which anthocyanins—compounds that impart the red color to wine—absorb light; in this sense, a greater presence of flavonols has been related to a visible effect on color [58].

Among the different flavonol metabolites identified in the ‘Cabernet Sauvignon’ variety, special mention should be given to isorhamnetin 3-sophoroside. This metabolite is a flavonoid-3-diglycoside containing a sophoroside moiety with a 1→2 glycosidic linkage to a carbohydrate moiety at the C3-position. There is limited literature available on isorhamnetin 3-sophoroside, and no reports of it in grapes or wine. This could make isorhamnetin 3-sophoroside a potential biomarker for the identification of ‘Cabernet Sauvignon’ wines made from ultrasound-treated grapes.

## 4. Conclusions

Untargeted metabolomics offers a powerful tool for studying the effects of sonication on the phytochemical composition of wine and for identifying potential markers of quality and/or process-induced changes. The findings of this research demonstrate the significant impact of high-power ultrasound treatment on the metabolite composition of red and white wines. The observed increase in metabolites, particularly in phenolic compounds such as hydroxycinnamic acids, stilbenes, and flavonols, suggests that ultrasound treatment effectively enhances the extraction of bioactive compounds from grape tissues during maceration. These changes contribute to the enhancement of wine quality attributes and, potentially, of the sensory characteristics of the final product. While the majority of research in this area has focused on red wine varieties, our study extended this investigation to white wine varieties, revealing similar trends in metabolite enrichment. This underscores the potential of high-power ultrasound as an innovative technology for improving the quality and diversity of wines across different grape varieties and styles. Overall, the results presented in this work contribute to advancing our knowledge of the application of ultrasound technology in winemaking and the use of untargeted metabolomics as an effective tool to discriminate between different types of ecological processes. Future research in this field could explore the long-term effects of ultrasound treatment on wine aging and stability and the consumer perception of wines produced using ultrasound technology, carrying out sensory evaluations, preference studies and consumer acceptance surveys.

## Figures and Tables

**Table 1:** List of tentative metabolites identified at statistically significantly increased levels in wines produced from sonicated white varieties compared to wines from non-sonicated grapes.

Rt (Min)	*m*/*z* Experimental	*m*/*z* Theoretical	Ion Annotation	Fragments *^b^*	Elemental Formula *^a^*	Tentative Metabolite Identification	Variety	References	Structure
***White wine***									
**5.70**	163.0395	163.0401	[M–H]^-^	145.0287; 119.0490	C_9_H_8_O_3_	Coumaric acid	A	YMDB00498	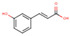
**5.87**	295.0455	295.0459	[M–H]^-^		C_13_H_12_O_8_	p-coutaric acid *	A	METLIN86490	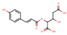
**5.18**	311.0402	311.0409	[M–H]^-^	179.0345; 161.0244	C_13_H_12_O_9_	Caftaric acid	A	YMDB01646	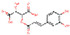
**5.70**	163.0395	163.0401	[M–H]^-^	145.0287; 119.0490	C_9_H_8_O_3_	Coumaric acid	Mac	YMDB00498	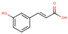
**5.87**	295.0455	295.0459	[M–H]^-^		C_13_H_12_O_8_	p-coutaric acid *	Mac	METLIN86490	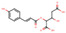
**10.62**	227.0708	227.0714	[M–H]^-^		C_14_H_12_O_3_	Resveratrol	Mac	Standard	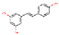
**7.37**	389.1235	389.1242	[M–H]^-^	227.0705	C_20_H_22_O_8_	Resveratrol 4’-glucoside	Mac	HMDB0030565	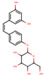
**6.38**	315.1078	315.1085	[M–H]^-^	153.0552	C_14_H_20_O_8_	Hydroxytyrosol 1-O-glucoside	Mac	HMDB0041024	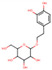

Metabolites were confirmed by comparison with pure standards, exact mass, isotopic pattern, fragments (20 ev of collision energy), libraries (Metlin, MoNA and Lipid Maps) and bibliography. Abbreviations: A, ‘Airén’; Mac, ‘Macabeo’. * Metabolites identified at level 3 according to the metabolomics consortium [31]. *^a^*: Formula from different libraries. *^b^*: Fragments are listed in the order of abundance in the spectrum.

**Table 2:** List of tentative metabolites identified at statistically significantly increased levels in wines produced from sonicated **red** varieties compared to wines from non-sonicated grapes.

Rt (min)	*m*/*z* Experimental	*m*/*z* Theoretical	Ion Annotation	Fragments *^b^*	Elemental Formula *^a^*	Tentative Metabolite Identification	Variety	References	Structure
***Red wine***									
**7.37**	389.1235	389.1242	[M–H]^-^	227.0705	C_20_H_22_O_8_	Resveratrol 4’-glucoside	CS	HMDB0030565	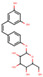
**10.62**	227.0708	227.0714	[M–H]^-^		C_14_H_12_O_3_	Resveratrol	CS	Standard	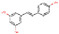
**9.12**	463.0875	463.0882	[M–H]^-^	301.0341	C_21_H_20_O_12_	Quercetin 3-galactoside	CS	YMDB01778	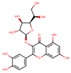
**9.83**	589.1205	589.1199	[M–H]^-^	301.0341	C_27_H_26_O_15_	Quercetin 3-(2″,3″,4″-triacetylgalactoside)	CS	METLIN50491	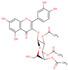
**8.15**	477.0670	477.0675	[M–H]^-^	301.0341	C_21_H_20_O_13_	Quercetin-3′-glucuronide	CS	YMDB01779	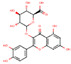
**14.05**	661.1555	661.1563	[M–H]^-^	285.0405	C_34_H_30_O_14_	Kaempferol 3-(2″,3″-diacetyl-4″-p-coumaroylrhamnoside)	CS	HMDB40537	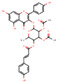
**9.01**	639.1560	639.1567	[M–H]^-^	315.0510	C_28_H_32_O_17_	Isorhamnetin 3-sophoroside	CS	LMPK12112373	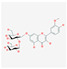
**8.88**	471.0925	471.0933	[M–H]^-^	333.0616; 289.0718; 183.0299	C_23_H_20_O_11_	(-)-Epigallocatechin 3-(4-methyl-gallate)	CS	HMDB0040293	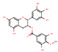
**8.50**	441.0820	441.0827	[M–H]^-^	289.0710; 169.0142	C_22_H_18_O_10_	(-)-Epicatechin 3-O-gallate	CS	HMDB0037944	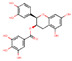
**6.57**	451.1250	451.1246	[M–H]^-^	289,0712; 151.0395	C_21_H_24_O_11_	Epicatechin 8-C-galactoside	CS	HMDB0039823	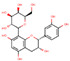
**6.57**	451.1250	451.1246	[M–H]^-^	289,0712; 151.0395	C_21_H_24_O_11_	Epicatechin 8-C-galactoside	S	HMDB0039823	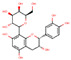

Metabolites were confirmed by comparison with pure standards, exact mass, isotopic pattern, fragments (20 ev of collision energy), libraries (Metlin, MoNA and Lipid Maps) and bibliography. Abbreviations: CS, ‘Cabernet Sauvignon’; S, ‘Syrah’. *^a^*: Formula from different libraries. *^b^*: Fragments are listed in the order of abundance in the spectrum.

## Data Availability

The data are contained within the article.
